# Polycipiviridae: a proposed new family of polycistronic picorna-like RNA viruses

**DOI:** 10.1099/jgv.0.000902

**Published:** 2017-08-31

**Authors:** Ingrida Olendraite, Nina I. Lukhovitskaya, Sanford D. Porter, Steven M. Valles, Andrew E. Firth

**Affiliations:** ^1^​ Division of Virology, Department of Pathology, University of Cambridge, Cambridge, CB2 1QP, UK; ^2^​ Center for Medical, Agricultural and Veterinary Entomology, USDA-ARS, 1600 SW 23rd Drive, Gainesville, FL 32608, USA

**Keywords:** Picornavirales, insect, RNA virus, ant, taxonomy

## Abstract

Solenopsis invicta virus 2 is a single-stranded positive-sense picorna-like RNA virus with an unusual genome structure. The monopartite genome of approximately 11 kb contains four open reading frames in its 5′ third, three of which encode proteins with homology to picornavirus-like jelly-roll fold capsid proteins. These are followed by an intergenic region, and then a single long open reading frame that covers the 3′ two-thirds of the genome. The polypeptide translation of this 3′ open reading frame contains motifs characteristic of picornavirus-like helicase, protease and RNA-dependent RNA polymerase domains. An inspection of public transcriptome shotgun assembly sequences revealed five related apparently nearly complete virus genomes isolated from ant species and one from a dipteran insect. By high-throughput sequencing and *in silico* assembly of RNA isolated from *Solenopsis invicta* and four other ant species, followed by targeted Sanger sequencing, we obtained nearly complete genomes for four further viruses in the group. Four further sequences were obtained from a recent large-scale invertebrate virus study. The 15 sequences are highly divergent (pairwise amino acid identities of as low as 17 % in the non-structural polyprotein), but possess the same overall polycistronic genome structure, which is distinct from all other characterized picorna-like viruses. Consequently, we propose the formation of a new virus family, Polycipiviridae, to classify this clade of arthropod-infecting polycistronic picorna-like viruses. We further propose that this family be divided into three genera: Chipolycivirus (2 species), Hupolycivirus (2 species) and Sopolycivirus (11 species), with members of the latter infecting ants in at least 3 different subfamilies.

## Abbreviations

Hel, helicase; IRES, internal ribosome entry site; LneV-1, Lasius neglectus virus 1; LniV-1, Lasius niger virus 1; MsaV-1, Myrmica scabrinodis virus 1; ORF, open reading frame; Pro, protease; RdRp, RNA-dependent RNA polymerase; SINV-2, Solenopsis invicta virus 2; SINV-4, Solenopsis invicta virus 4; SNP, single nucleotide polymorphism; TSA, transcriptome shotgun assembly.

## Introduction

The order *Picornavirales* currently comprises the families *Dicistroviridae*, *Iflaviridae*, *Marnaviridae*, *Picornaviridae* and *Secoviridae*, and the unassigned genera *Bacillarnavirus* and *Labyrnavirus*. Members of the order are characterized by (i) a positive-sense RNA genome, usually with a 5′ covalently linked VPg (virus protein, genome linked) and a 3′ poly(A) tail, (ii) a polyprotein gene expression strategy with cleavage mainly mediated by viral protease(s), (iii) a structural protein module containing three jelly-roll capsid protein domains which form small non-enveloped icosahedral virions with pseudo *T*=3 symmetry and (iv) a non-structural protein module containing a superfamily III helicase (or NTPase), a 3C-like chymotrypsin-like protease and a superfamily I RNA-dependent RNA polymerase (RdRp), encoded sequentially in that order [1]. In contrast, the families *Caliciviridae* and *Potyviridae* (for example), although termed picorna-like (and informally grouped into a ‘picorna-like superfamily’) are excluded from the *Picornavirales* order for various reasons; for example, caliciviruses encode a single jelly-roll capsid protein and virions have true *T*=3 symmetry, while potyviruses have an unrelated capsid protein and filamentous virions, and encode a superfamily II instead of a superfamily III helicase.

Solenopsis invicta virus 2 (SINV-2) is a positive-sense single-stranded RNA virus that infects the red imported fire ant, *Solenopsis invicta* Buren, an invasive species in the southern USA [2]. Replicating virus was detected in the larval and adult stages of *S. invicta* [3]. While field infection rates are rather low for *S. invicta* [4, 5], there are distinct fitness costs for founding queens infected with the virus [6]. Colonies established from SINV-2-infected queens produced less brood and had longer claustral periods. SINV-2 infection was also associated with significant up-regulation of global gene expression in *S. invicta* queens, including immune response genes [6]. The monopartite genome was originally found to contain three open reading frames (ORFs) in its 5′ third and a single long ORF in its 3′ two-thirds, though we show here that there are actually four consecutive 5′ ORFs, one having been overlooked previously as a result of a sequencing error. The polypeptide encoded by the 3′ ORF contains motifs characteristic of a superfamily III helicase, a 3C-like chymotrypsin-related protease and a superfamily I RdRp [2]. A phylogenetic analysis of RdRp sequences placed SINV-2 within the ‘picorna-like superfamily’, but outside of established virus families [7]. Isometric particles with a diameter of ~33 nm were only found in ants testing positive for SINV-2 by RT-PCR [2].

By searching the NCBI transcriptome shotgun assembly (TSA) database, we identified six related sequences, of which five were derived from ant samples. To further explore the diversity and prevalence of this group of viruses, we performed high-throughput sequencing of RNA derived from *Solenopsis invicta* and four UK ant species. This led to the identification of four additional viruses. During the course of this work, 1445 new viruses were identified via high-throughput sequencing [8], of which four exhibited similar genome organizations to SINV-2. We performed a phylogenetic and comparative genomic analysis of these SINV-2-like virus sequences. All have a characteristic genome organization, with four consecutive 5′ proximal ORFs and one long 3′ ORF. ORFs 1, 3 and 4 are predicted to encode jelly-roll fold capsid proteins, while ORF2 encodes a protein of unknown function. Ant-infecting members of the group apparently have an additional 5′ ORF (ORF2b) overlapping the 5′ end of ORF2 and encoding a small protein containing a predicted transmembrane domain. The 3′ ORF5 encodes helicase, protease and RdRp domains, is presumed to encode a VPg and potentially another protein between the helicase and protease, and is likely to encode one or more additional proteins upstream of the helicase. The unusual genome organization and phylogenetic distinctness of this group of viruses suggests they should be classified into a new virus family, for which we propose the name Polycipiviridae (**polyci**stronic **pi**corna-like viruses). The characteristic complement of picornavirus-like protein domains suggests that the Polycipiviridae should be included within the *Picornavirales* order.

## Results

### Identification of SINV-2-like viruses

**Fig. 1.**
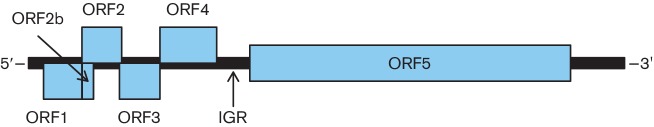
Genome map of SINV-2. The genome is represented by a black line. ORFs are indicated in pale blue with vertical offsets indicating the reading frame (−1, 0, +1) relative to ORF5. IGR, intergenic region.

The SINV-2 genome was first sequenced by Valles *et al*. [2]. Suspicious of a large apparently non-coding gap following ORF1, we resequenced the SINV-2 genome. This resulted in the correction of a UGA stop codon to a UGG tryptophan codon, after which it became apparent that the region following ORF1 is actually occupied by an ORF. The reannotated SINV-2 ORFs are shown in Fig. 1. The new SINV-2 sequence has been deposited in GenBank under accession MF041813.1, and has 143 nucleotide differences relative to the previous SINV-2 sequence EF428566.1.

**Fig. 2.**
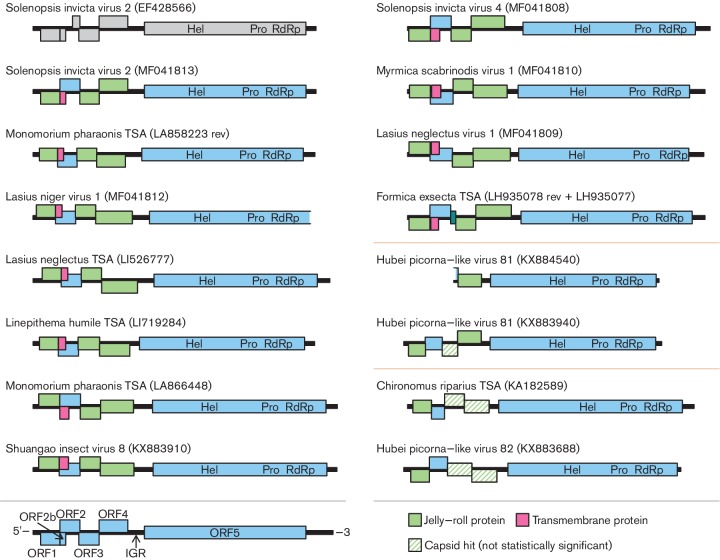
Genome maps of proposed polycipiviruses. ORFs are represented by coloured rectangles with vertical offsets indicating reading frames relative to the Hel-Pro-RdRp ORF. Green rectangles indicate ORFs encoding jelly-roll capsid protein domains as predicted by HHpred; hatched green lines indicate uncertain capsid domain identifications. Pink rectangles indicate a conserved overlapping ORF encoding a transmembrane helix as predicted by TMHMM. A dark green rectangle in the *F. exsecta* TSA map indicates an additional ORF, 3a. Replication protein domains, identified by their conserved characteristic motifs, are indicated as Hel (helicase), Pro (protease) and RdRp (RNA-dependent RNA polymerase). TSA indicates sequences obtained from the NCBI Transcriptome Shotgun Assembly database. Two horizontal lines (on the right side) separate the three proposed genera.

Further high-throughput sequencing of *Solenopsis invicta* RNA samples revealed the presence of a new SINV-2-like virus, which we named Solenopsis invicta virus 4 (SINV-4). A complete genome was obtained by Sanger sequencing and has been deposited in GenBank with the accession number MF041808.1. SINV-4 has the same genome organization (Fig. 2), and 31 % amino acid identity to SINV-2 in the ORF5 polypeptide.

Next we queried the NCBI Transcriptome Shotgun Assembly (TSA) database using tblastn with the SINV-2 ORF5 polypeptide as the query and eukaryotes as the target taxonomic group. Amongst the top matching ‘hits’, long sequences (>10 kb) were identified, and their longest ORF extracted, translated and queried against NCBI RefSeq virus genome sequences. Sequences whose reciprocal best match was SINV-2 were retained, while other sequences (typically with top matches to iflavirus or dicistrovirus sequences) were discarded. This resulted in five long sequences with reciprocal best matches to SINV-2. An additional long sequence was obtained by merging the partly overlapping fragments LH935078.1 and LH935077.1. Five of the long sequences derive from ant RNA-Seq libraries (order Hymenoptera, family Formicidae; LA858223.1 and LA866448.1 from *Monomorium pharaonis*, LI526777.1 from *Lasius neglectus*, LI719284.1 from *Linepithema humile* and the combined LH935078.1+LH935077.1 from *Formica exsecta*). The sixth sequence, KA182589.1, derives from a *Chironomus riparius* RNA-Seq library (order Diptera; family Chironomidea). Sequence attributes are recorded in Table 1 and the genome organizations are depicted in Fig. 2. A number of shorter partial genome sequences were also identified, but are not discussed further here.

**Table 1.** Viruses in the proposed family Polycipiviridae

Sequence name	Accession no.	Host species or source	Sequence length§, nt	5′ UTR length||	ORF coordinates¶													3′ UTR length||
					ORF1		ORF2b		ORF2		ORF3		ORF4		IGR length	ORF5		
					Start	End	Start	End	Start	End	Start	End	Start	End		Start	End	
SINV-2	EF428566.1 MF041813.1	*Solenopsis invicta*	11303**	301	302	1078	1079	1309	1075	1878	1871	2647	2644	3792	662	4455	10916	387
*M. pharaonis* TSA	LA858223.1 rev	*Monomorium pharaonis*	11259**	255	256	984	985	1221	981	1763	1753	2547	2544	3692	658	4351	10812	447
LniV-1	MF041812.1	*Lasius niger*	11092	134	135	899	900	1157	896	1705	1695	2507	2507	3970	687	4658	–	–
*L. neglectus* TSA	LI526777.1	*Lasius neglectus*	11854**	366	367	1131	1132	1392	1128	1940	1930	2742	2742	4181	657	4839	11375	479
*L. humile* TSA	LI719284.1	*Linepithema humile*	11245**	258	259	1011	1015	1305	1011	1817	1804	2664	2661	3872	385	4258	10860	385
*M. pharaonis* TSA	LA866448.1	*Monomorium pharaonis*	12160**	230	231	1064	1069	1434	1065	1898	1891	2697	2702	4018	759	4778	11719	441
Shuangao insect virus 8	KX883910.1	Insects mix (1)†	12155	224	225	1046	1050	1415	1046	1882	1875	2693	2690	4033	768	4802	11755	400
SINV-4	MF041808.1	*Solenopsis invicta*	12077**	198	199	1065	1069	1434	1065	1898	1891	2697	2697	4034	734	4769	11662	415
MsaV-1	MF041810.1	*Myrmica scabrinodis*	11872**	228	229	1062	1111	1482	1059	1973	1970	2773	2776	4134	438	4573	11430	442
LneV-1	MF041809.1	*Lasius neglectus*	11821**	207	208	1053	1099	1422	1050	1946	1943	2782	2772	4259	336	4596	11402	419
*F. exsecta* TSA	LH935078.1 rev LH935077.1	*Formica exsecta*	11899	206	207	1046	1080	1388	1043	1876#	2079	2879	2882	4300	386	4687	11535	364
Hubei picorna-like virus 81	KX884540.1	*Procambarus clarkia*	8116	–	–	–	–	–	–	–	–	84	81	1019	357	1377	8006	110
Hubei picorna-like virus 81	KX883940.1	Insects mix (2)‡	10315	189	190	867	–	–	857	1540	1537	2157	2154	3098	353	3452	10078	237
*Ch. riparius* TSA	KA182589.1	*Chironomus riparius*	11462	365	366	1109	–	–	1109	1627	1630	2418	2421	3413	384	3798	11144	318
Hubei picorna-like virus 82	KX883688.1	Spiders mix*	11081	279	280	1023	–	–	1023	1751	1748	2722	2719	3723	442	4166	10939	142

*Spiders mix (*Neoscona nautica*, *Parasteatoda tepidariorum*, *Plexippus setipes*, *Pirata* sp., Araneae sp.).

†Insects mix (1) (*Abraxas tenuisuffusa*, *Hermetia illucens*, Chrysopidae sp., Coleoptera sp., *Psychoda alternata*, Diptera sp., Stratiomyidae sp.).

‡Insects mix (2) (*Paramercion melanotum*, *Paracercion calamorum*, *Ceriagrion auranticum*, *Brachydiplax chalybea*, *Orthetrum albistylum*, *Pseudothemis zonata*, *Chironomus* sp.).

§Excluding polyA (if present).

||UTRs are likely to be incomplete in some species.

¶ORF coordinates (including stop codon).

#The *F. exsecta* TSA sequence has an additional ORF (ORF3a; 1873 - 2082) between ORF2 and ORF3.

**Sequence extends to 3′ polyA.

Given that SINV-2-like viruses appeared to be most prevalent in ants, we obtained samples from four different ant species (*Lasius flavus*, *L. niger*, *L. neglectus* and *Myrmica scabrinodis*) collected in Cambridge, UK, and performed two rounds of high-throughput RNA sequencing. Contigs were assembled using Trinity [9, 10] and Velvet [11], and SINV-2-like sequences were identified with blastx.

In the first round, we sequenced *L. niger* and *L. flavus* RNA samples using both small-RNA sequencing to enrich for 21–22 nt virus-derived RNAs that are expected to be produced as a result of the insect RNA interference (RNAi) anti-viral defence pathway, and standard RNA-Seq. In this case, good contig assemblies were not obtained for the small-RNA samples and no further small-RNA sequencing was performed. For the long RNA-Seq of *L. niger*, but not *L. flavus*, we identified one SINV-2-like sequence. In the second round, we performed long RNA-Seq for RNA obtained from *M. scabrinodis*, *L. neglectus* and a second *L. flavus* sample, and identified SINV-2-like sequences for *M. scabrinodis* and *L. neglectus*. Morphological ant species identifications were confirmed by blastn of cytochrome C oxidase subunit I (COI) sequences obtained from the transcriptome assemblies against the NCBI non-redundant (nr) nucleotide database.

The three SINV-2-like sequences were verified by Sanger sequencing and extended where possible by 5′ and 3′ RACE (see below). Sequence attributes are recorded in Table 1 and the genome organizations are depicted in Fig. 2. Hereafter we refer to these viruses as Lasius niger virus 1 (LniV-1), Lasius neglectus virus 1 (LneV-1) and Myrmica scabrinodis virus 1 (MsaV-1), with the GenBank accession numbers MF041812.1, MF041809.1 and MF041810.1, respectively.

**Fig. 3.**
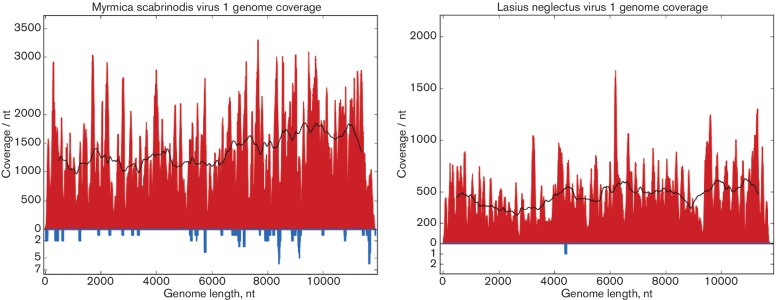
RNA sequencing coverage of the MsaV-1 and LneV-1 genomes. The total coverage at each nucleotide position is indicated in red (positive sense) or blue (negative sense; note the different axis scale). Black lines show the mean coverage in a 1000-nt sliding window.

Using Bowtie 2 [12], we mapped RNA-Seq reads (>30 nt) back to the assembled virus genomes to assess coverage and variation. LniV-1, LneV-1 and MsaV-1 had mean coverage values of 6.4, 457 and 1348, respectively. For the two viruses with high coverage (Fig. 3), we identified single nucleotide polymorphisms (SNPs) that were present in >10 % of reads. In MsaV-1, we found seven SNPs in coding regions, all non-synonymous and with frequencies just over 10 %. In LneV-1, we found 87 SNPs, of which 62 were synonymous – 5/22 in ORF1, 3/3 in ORF2, 7/7 in ORF3, 8/8 in ORF4 and 39/47 in ORF5.

During the course of the above work, 1445 new RNA viruses were identified via high-throughput sequencing of RNA samples obtained from a variety of invertebrates [8]. The authors identified the most similar previously published virus sequence for each new virus, four of which were found to have highest similarity to SINV-2. These four sequences derive not from ants, but from a mixed sample of spiders (KX883688.1), a mixed insect sample including members of the orders Odonata and Diptera (KX883940.1), the crayfish *Procambarus clarkia* (KX884540.1) and a mixed insect sample including members of the orders Lepidoptera, Diptera, Neuroptera and Coleoptera (KX883910.1), with respectively 19, 21, 21 and 30 % amino acid sequence identity to SINV-2 in the ORF5 polypeptide. The sequences from spiders, Odonata/Diptera and crayfish have low abundance (<0.1 % of sample non-rRNA), leading the authors to suggest the possibility that they may derive from parasites or gut contents (which in these cases might include insects) instead of the target organisms. Sequence attributes are recorded in Table 1 and the genome organizations are depicted in Fig. 2.

In summary, we identified a total of 15 SINV-2-like sequences (Table 1), comprising a correction of the original SINV-2 sequence, SINV-4, six sequences from the NCBI TSA database, three sequences from UK ant species and four additional recently published sequences. Fourteen of the sequences appear to represent complete or nearly complete virus genomes.

### Genome organization of SINV-2-like viruses

**Fig. 4.**
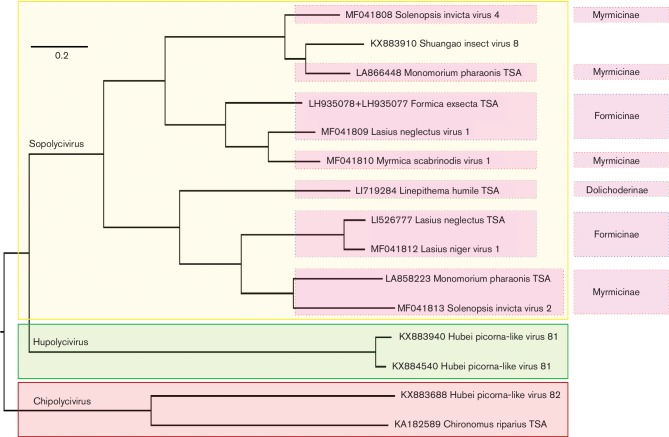
Phylogenetic tree of the proposed family Polycipiviridae. ORF5 amino acid sequences were aligned with muscle, and a Bayesian Markov chain Monte Carlo-based phylogenetic tree was produced with MrBayes. All posterior probabilities were equal to 1.00. The tree was mid-point rooted and visualized with FigTree. TSA indicates sequences obtained from the NCBI Transcriptome Shotgun Assembly database. Proposed Polycipiviridae genera – Chipolycivirus, Hupolycivirus and Sopolycivirus – are delineated with boxes. For sopolyciviruses, the host ant subfamilies are indicated on the right-hand side.

Each full-length sequence had five main ORFs (Fig. 2). The stop and start codons of consecutive ORFs in the 5′ region have closely spaced (often overlapping) stop and start codons (Table 1). We also identified a shorter sixth ORF (termed ORF2b) in some members of the group (Fig. 2). ORF2b overlaps the 5′ end of ORF2 in the +1 frame relative to ORF2. The *Formica exsecta* TSA contains an additional short ORF (ORF3a) between ORFs 2 and 3. Hubei picorna-like virus 81 (accession number KX884540; [8]) appears to be incomplete, lacking ORFs 1 and 2 and most of 3. Sequences with ORF2b cluster phylogenetically (Fig. 4; see below) and all are ant-associated, except for Shuangao insect virus 8, which was derived from an ‘insect mix’ that was not reported to contain members of the family *Formicidae* [8]. However, insects within this sample may include predators of ants; thus the sequence could potentially have originated from an ant (or other) host.

Most of the 15 sequences had some amount of the 5′ and 3′ untranslated regions (UTRs) present, though, given the variability in UTR lengths, we assume that several were incomplete. The 5′ UTR sequences were frequently >200 nt and ranged up to 366 nt in length. Eight sequences extend to the poly(A) tail and may therefore be assumed to have been 3′-complete. For these sequences, the 3′ UTR lengths ranged from 385 to 479 nt, excluding the poly(A) sequence. The length of the intergenic region between ORFs 4 and 5 ranged from 336 to 768 nt. To assess 5′-completeness we compared 5′ nucleotide sequences between different species (Figure S1, available in the online Supplementary Material). A number of polycipivirus sequences exhibit a predicted stable simple stem–loop structure close to the 5′ end. In SINV-4, SINV-2 and the *L. neglectus* TSA, this is preceded by an AU-rich tract with 5′-terminal UUU. This 5′ end similarity between these highly divergent sequences suggests that this represents the true 5′ end of the genome. Relative to these, the MsaV-1 and LneV-1 sequences appear to be missing approximately 5 and 23 nt, respectively, from their 5′ ends. For the LniV-1 sequence we were unable to obtain complete 5′ or 3′ UTR sequences.

For each ORF, functions were predicted with HHpred [13] using the Pfam [14] and PDB [15] databases. For most sequences, ORFs 1, 3 and 4 were predicted to encode picornavirus-like capsid proteins (Fig. 2). For the most divergent sequences – Hubei picorna-like viruses 81 and 82, and the *C. riparius* TSA – ORF1 was predicted to encode a picornavirus-like capsid protein, but for ORF4 this was only predicted for Hubei picorna-like virus 81, and for ORF3 it was not predicted for any of the three. However, for ORFs 3 and 4, HHpred [16, 17] found significant sequence alignments (E-values <0.0001; alignment lengths ranging from 112 to 245 amino acids) between an alignment of the 11 sequences from the SINV-4/SINV-2 clade and a query alignment of the *C. riparius* TSA and Hubei picorna-like virus 82, or the single Hubei picorna-like virus 81 sequence, suggesting that these ORFs encode homologous proteins across all 14 sequences. For all sequences, ORF5 was predicted to encode helicase (Hel), protease (Pro) and RNA-dependent RNA polymerase (RdRp) domains. ORFs 2 and 2b had no HHpred matches; however, a transmembrane domain was predicted using TMHMM [18] in the middle of all ORF2b amino acid sequences. HHpred indicated homology between ORF2 of the SINV-4/SINV-2 clade and ORF2 of Hubei picorna-like virus 81 (E-value <10^−8^; alignment length 126 amino acids), but homology to ORF2 of the *C. riparius* TSA/Hubei picorna-like virus 82 clade was less certain (E-value=0.029; alignment length 165 amino acids). The *Formica exsecta* TSA ORF3a polypeptide also had no significant HHpred matches.

Following Koonin and Dolja [19], we identified characteristic Hel, Pro and RdRp motifs in the ORF5 polypeptide sequences. All sequences contained the three superfamily III helicase motifs (Fig. S2). The protease domain is less well conserved across picorna-like viruses, with only three very short characteristic motifs corresponding to the catalytic triad – H, D and C (within a quite conserved GxCG motif) [19]. Positive-sense RNA viruses, and in particular picorna-like viruses, usually have chymotrypsin-like cysteine proteases. In the SINV-2-like sequences, however, the protease has a serine (S) at the corresponding active site, GxSG (Fig. S3). We were able to find all eight conserved RdRp motifs (Fig. S4), with the motifs most closely matching superfamily I RdRps (a group that also includes the RdRps of picornaviruses, potyviruses, sobemoviruses and nidoviruses [19]). In two sequences (the *C. riparius* TSA and Hubei picorna-like virus 82), the usually very well-conserved GDD in motif VI (also often called motif C) was replaced with ADD.

### Phylogeny of SINV-2-like viruses

**Fig. 5.**
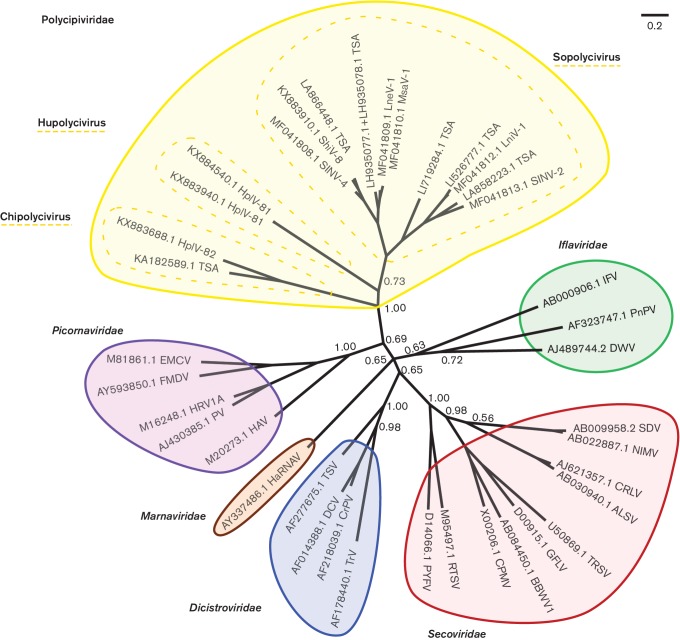
Phylogenetic tree for polycipiviruses and representative members of the order *Picornavirales*. Core RdRp amino acid sequences from representative *Picornavirales* viruses were obtained from Koonin *et al.* [7] and combined with the equivalent regions from the 15 sequences in Table 1 with RdRp coverage. Sequences were aligned with muscle, and a Bayesian Markov chain Monte Carlo-based phylogenetic tree was produced. Posterior probabilities are indicated for family root nodes, and elsewhere if *P*<1.00. Abbreviations: ALSV, apple latent spherical virus; BBWV1, broad bean wilt virus 1; CPMV, cowpea mosaic virus; CRLV, cherry rasp leaf virus; CrPV, cricket paralysis virus; DCV, Drosophila C virus; DWV, deformed wing virus; EMCV, encephalomyocarditis virus; FMDV, foot-and-mouth disease virus; GFLV, grapevine fanleaf virus; HaRNAV, Heterosigma akashiwo RNA virus; HAV, hepatitis A virus; HplV-81, Hubei picorna-like virus 81; HplV-82, Hubei picorna-like virus 82; HRV1A, human rhinovirus 1A; IFV, infectious flacherie virus; LneV-1, Lasius neglectus virus 1; LniV-1, Lasius niger virus 1; MsaV-1, Myrmica scabrinodis virus 1; NIMV, navel orange infectious mottling virus; PnPV, Perina nuda picorna-like virus; PV, polio virus; PYFV, parsnip yellow fleck virus; RTSV, rice tungro spherical virus; SDV, satsuma dwarf virus; ShiV-8, Shuangao insect virus 8; SINV-2, Solenopsis invicta virus 2; SINV-4, Solenopsis invicta virus 4; TRSV, tobacco ringspot virus; TrV, Triatoma virus; TSV, Taura syndrome virus.

Based on the genome structure and the identified protein domains, SINV-2-like sequences may be classified within the order *Picornavirales*. To test for monophyly, we obtained amino acid sequences for the conserved ‘core’ region of the RdRp from viruses in the order *Picornavirales* from the supplementary material of [7], appended the equivalent region from the 15 SINV-2-like sequences with RdRp coverage, realigned the sequences with muscle [20] and generated a Bayesian Markov chain Monte Carlo-based phylogenetic tree using MrBayes [21]. The SINV-2-like sequences form a monophyletic (albeit highly divergent) group (Fig. 5).

## Discussion

We have identified and sequenced four new viruses from ant species. Together with SINV-2, 6 sequences recovered from TSA databases and 4 recent additions to GenBank, these 15 sequences form a distinct group of arthropod-infecting viruses with a characteristic genome organization. Members have a polyadenylated positive-sense RNA genome that encodes three related picornavirus-like jelly-roll capsid domains, and a non-structural polyprotein containing superfamily III helicase, 3C-like chymotrypsin-related protease and superfamily I RdRp domains, characteristic of members of the order *Picornavirales*. Thus we propose that they should be classified into a new family, Polycipiviridae (**polyci**stronic **pi**corna-like viruses), within the order *Picornavirales*. The virion morphology (small icosahedral particles) observed for SINV-2 [2] is also consistent with this placement. Like the dicistronic dicistroviruses (family *Dicistroviridae*) and the bipartite cheraviruses, sadwaviruses and comoviruses (family *Secoviridae*), the polycipivirus coding sequences are split into separate non-structural and structural protein modules. In contrast to these other groups, the polycipivirus structural protein module is further split into separate 5′ ORFs rather than depending on polyprotein expression of multiple jelly-roll domains from a single ORF.

Although polycipiviruses form a distinct clade, there is considerable diversity among the 15 available sequences (some of the ORF5 pairwise amino acid identities are as low as 17 %), indicating that the family should be split into a number of genera. We propose the following groupings of the currently available sequences (see Fig. 4): Sopolycivirus (from **So**lenopsis invicta **polyci**pivirus) to include SINV-2, SINV-4 and nine related sequences, all of which contain ORF2b; Hupolycivirus (from **Hu**bei picorna-like virus 81 **polyci**pivirus) to include the two Hubei picorna-like virus 81 sequences; and Chipolycivirus (from **Chi**ronomus riparius **polyci**pivirus) to include the *C. riparius* TSA and Hubei picorna-like virus 82, both of which contain the GDD to ADD substitution in RdRp motif VI.

The polycipivirus 3C-like protease is unusual in that it contains a serine residue at its active site (serine protease), whereas the majority of *Picornavirales* 3C-like proteases have a cysteine residue at this location (cysteine protease). The only other currently known exceptions are the sole member of the *Picornavirales* family, *Marnaviridae*, and some members of genus *Nepovirus* in the family *Secoviridae* [1, 22]. The ‘picorna-like’ astroviruses, sobemoviruses and poleroviruses also have chymotrypsin-like serine proteases, and cellular homologues have serine at the active site [23, 24].

The most conserved protein of positive-sense RNA viruses is the RdRp. In polycipiviruses, we were able to identify all eight signature motifs of the superfamily I RdRp. Unusually, however, in two of the sequences the highly conserved GDD of motif VI was replaced with ADD. Motif VI is responsible for magnesium ion coordination. The first aspartate (D) is mainly responsible for the coordination and cannot be substituted. There is, however, potential for flexibility in the third position, and even more flexibility in the first position (reviewed in [25]). Indeed, the glycine (G) has been experimentally substituted with six different amino acids, and with some substitutions the polymerase remains active. Substitution with alanine (ADD) in tobacco vein mottling virus (family *Potyviridae*), polio virus (family *Picornaviridae*) or hepatitis C virus (family *Flaviviridae*) results in an *in vitro* RdRp activity of 5 to 12 % of wild-type activity [26–28], although when the same mutation was introduced in encephalomyocarditis virus (family *Picornaviridae*) the RdRp was inactive *in vitro* [29]. Positive-sense and double-stranded RNA viruses have a strong preference for GDD, although nidoviruses (order Nidovirales) and some, but not all, hypoviruses (family *Hypoviridae*) have SDD at this site. On the other hand, non-segmented negative-sense RNA viruses generally have GDN, whereas segmented negative-sense RNA viruses have SDD, and the reverse transcriptases of retroviruses have MDD [30–33]. Unusually, members of the positive-sense RNA virus family *Permutotetraviridae* and the double-stranded RNA virus family *Birnaviridae* have a permuted polymerase domain, with motif VI occurring between motifs III and IV; the GDD sequence is still present in permutotetraviruses but is substituted with ADN in birnaviruses, and mutation of ADN to GDD results in an almost complete loss of activity [34, 35]. In summary, therefore, even though the ADD in motif VI of two polycipivirus sequences is a deviation from the expected GDD, it is still a plausible variation. The fact that it was observed in two independent sequences (which also cluster phylogenetically and are relatively distant from the other polycipivirus sequences; Fig. 4) indicates that ADD is a real variant and not a result of sequencing error.

Where investigated, *Picornavirales* species have been found to harbour a VPg protein covalently linked to the 5′ end of their genome that primes RNA synthesis during genome replication. The *Picornavirales* VPg is typically <5 kDa and normally encoded between Hel and Pro [1]. Due to their small size and lack of structural domains, divergent VPg proteins are not easily recognizable by sequence homology, and we were not able to definitively identify a VPg domain in polycipivirus sequences. Nonetheless, there is a large region of unassigned function between Hel and Pro that we presume encodes a VPg, and probably (due to the size of the region) another protein of unknown function. A further one or two additional proteins are likely encoded upstream of Hel in the ORF5 polyprotein. Due to the high divergence between polycipiviruses and picorna-like viruses whose cleavage sites have been characterized, we were unable to definitively predict the polyprotein cleavage sites.

As in other *Picornavirales* species, gene expression in polycipiviruses likely depends on internal ribosome entry site (IRES)-mediated initiation. Consistent with this, the lengthy 5′ UTRs typically contained a number of AUG codons that would be expected to inhibit 5′ end-dependent scanning. Consequently, we suppose the 5′ UTR to contain an IRES to direct ribosome initiation at the ORF1 start codon. Similarly, we suppose the long intergenic region between ORFs 4 and 5 to contain a second IRES to direct ribosome initiation at the ORF5 start codon. A similar situation occurs in dicistroviruses (family *Dicistroviridae*), where the translation of structural and non-structural polyproteins is directed by separate IRESes (although in that case the non-structural polyprotein ORF is 5′ proximal). Although we attempted to define the potential IRES RNA structure *in silico* by means of RNA-folding algorithms and inter-species comparisons, the results to date have been inconclusive. The translation mechanism of the additional 5′ ORFs (ORFs 2 to 4 and, where present, 2b) remains uncertain; however, close spacing of the stop and start codons of consecutive ORFs suggests a ribosome reinitiation mechanism.

Additional work will be required to confirm the composition and structure of virus particles, the presence and sequence of a 5′-linked VPg protein, the non-structural polyprotein cleavage sites and products, and the gene expression mechanisms of this unusual family of arthropod-infecting picorna-like viruses.

Ten of the 11 members of the proposed Polycipiviridae genus Sopolycivirus appear to infect ant species, while the eleventh member (Shuangao insect virus 8) is apparently an insect virus whose host has yet to be identified (Fig. 4). These viruses have been isolated from ants across several continents and in four out of the five ant species targeted in this study. Moreover, they have been identified in three different ant subfamilies, and three individual ant species (*Solenopsis invicta*, *Monomorium pharaonis* and *Lasius neglectus*) were found to play host to more than one divergent viruses in the group, suggesting a long evolutionary history between Sopolycivirus species and ants. Although we cannot rule out other hosts, and our own sampling has been biased towards discovering new ant-infecting members, it seems possible that genus Sopolycivirus may be an ant-specific clade.

## Methods

### SINV-4 identification and sequencing

Adult worker *Solenopsis invicta* ants were collected from 46 nests in the eastern portion of the state of Formosa, Argentina, and returned to quarantine in Gainesville, Florida, United States. Total RNA was extracted from 10 to 15 live worker ants from each colony using Trizol (Invitrogen) and the PureLink purification kit (Ambion). Total RNA (10 µg per group) was submitted to GE Healthcare (Los Angeles, CA, USA) for mRNA purification, library preparation and Illumina RNA sequencing (MiSeq). Sequences were aligned to the *S. invicta* genome (GenBank accession AEAQ01000001.1) and non-matching sequences were compared to the UniProt annotated Swiss-Prot protein sequence database (download date 14 November 2014). The unmatched sequences were assembled (Vector NTI, Invitrogen) and a unique sequence with significant similarity to SINV-2 was identified by blastx analysis. RT-PCR with gene-specific oligonucleotide primers revealed that this sequence was present in some colonies of *S. invicta* in the USA. Total RNA from USA *S. invicta* colonies containing the sequence was extracted and used as template for cDNA synthesis, subsequent PCR and RACE (5′ and 3′) to obtain the entire genome sequence of this new virus, SINV-4, by Sanger sequencing. For 5′ RACE, the 5′ RACE System for Rapid Amplification of cDNA Ends, version 2.0 (Invitrogen, Carlsbad, CA, USA) was used. cDNA was first synthesized with oligonucleotide p1476 (5′-TGGAATTCCAGAATTTTCTAAGGTTCCCATATTAGT), followed by PCR with the gene-specific primer p1474 (5′-TGAATTCCAGGTAACGCTTGAACCATTGGT) and the abridged anchor primer (Invitrogen). For 3′ RACE, the GeneRacer kit (Invitrogen) was used. cDNA was synthesized with the GeneRacer Oligo dT primer. PCR was subsequently completed with the GeneRacer 3′ primer and the gene-specific primer, p1536 (5′-ATGGCTGTTGCTGACATGTTATG CATTATGTT).

### LniV-1, LneV-1 and MsaV-1 identification and sequencing

Adult *L. niger*, *L. neglectus*, *M. scabrinodis* and *L. flavus* ants were collected from individual nests in Cambridge, UK; all ants were workers except the sequencing round 1 *L* *. flavus* sample, for which queens were used. Total RNA was extracted from 10 to 20 adult workers or 5 queens using Trizol (Invitrogen) following the manufacturer’s instructions. RNA was treated with DNase (Promega, RQ1 RNase-free DNase). For standard RNA-Seq libraries, ribosomal RNA was depleted using the Ribo-Zero kit (Illumina), and the remaining RNA subjected to alkaline hydrolysis, followed by acrilamide gel purification of RNA bands within the size range 35–50 nt for *L. niger* and 70–85 for the other four species. For small-RNA sequencing, RNA bands within the size range 18–30 nt were acrylamide gel-purified from total RNA after DNase treatment. All library amplicons were constructed using a small RNA cloning strategy [36, 37] and sequenced (single-end, 75 nt) using the NextSeq500 platform (Illumina) at the DNA Sequencing Facility (Department of Biochemistry, University of Cambridge). High-throughput sequencing data were deposited in ArrayExpress (http://www.ebi.ac.uk/arrayexpress) under the accession number E-MTAB-5781. RNA-Seq reads were trimmed using the FASTX-Toolkit and assembled using the Trinity (v 2.3.2) and Velvet (v 1.2.10) *de novo* assemblers [10, 11]. Using blastx, the assembled contigs were compared to a database of polypeptide sequences derived from SINV-2, SINV-4 and the SINV-2-like sequences identified in the NCBI TSA database. Contigs that mapped to SINV-2-like proteins with E-value <10^−6^ and length >300 nt of coding sequence were retained, manually joined where possible, and used to design primers for Sanger sequencing.

Gaps were filled and the entire genomes sequenced by Sanger sequencing and terminal sequences obtained by 5′ and 3′ RACE, as follows. Two µg of total ant RNA was treated with proteinase K and then recovered by acid phenol/chloroform extraction and used for 5′- and 3′-RACE using the SMARTer RACE 5′/3′ kit (Clontech) according to the manufacturer's instructions. Gene-specific primers for 5′ and 3′ RACE were located within 1000 nt of the expected 5′ and 3′ ends of the virus genome. The resulting clones (12–19 for each of MsaV-1 5′ and 3′, LneV-1 5′ and 3′ and LniV-1 5′, and 4 for LniV-1 3′) in the pRACE vector were sequenced with M13 universal primers. The rest of the viral genomes were PCR-amplified as eight partially overlapping DNA fragments using specific primers and the same cDNA templates as were generated for the 5′/3′ RACE PCRs. These fragments were cloned into the pJET1.2 vector (Thermo Scientific) and sequenced with pJET1.2-specific primers.

### Computational analysis

Sequences were processed using EMBOSS [38] and analysed using blast [39], HHpred [13] (using the PDB and Pfam databases) and Phyre 2 [40] (analysis performed between November 2016 and May 2017). Comparison of amino acid sequences/alignments between different polycipivirus clades was performed using HHpred in the align two sequences/alignments mode during 23–28 June 2017. Amino acid sequences were aligned using muscle v 3.8.31 [20] and phylogenetic trees were estimated using the Bayesian Markov chain Monte Carlo method implemented in MrBayes v 3.2.6 [21], sampling across the default set of fixed amino acid rate matrices, with 10 million generations, and discarding the first 25 % as burn-in. The trees were visualized using FigTree v 1.4.3. Pairwise amino acid identities were calculated based on pairwise ORF5 muscle alignments. To calculate the coverage and polymorphism frequencies, Bowtie 2 (v 2.3.0, [12]), using default parameters, was used to map raw NextSeq sequencing reads back to the LniV-1, LneV-1 and MsaV-1 genomes.

### Accession numbers, vouchers, and new family submission

We have submitted a proposal to the International Committee on Taxonomy of Viruses (ICTV) to name a new family Polycipiviridae containing the genera Sopolycivirus, Hupolycivirus and Chipolycivirus in the order *Picornavirales*. Sequences for SINV-4, SINV-2, LneV-1, MsaV-1 and LniV-1 have been submitted to NCBI with the accession numbers MF041808, MF041813, MF041809, MF041810 and MF041812. Voucher specimens of the *S. invicta* ant species from which SINV-4 was originally sequenced are retained at USDA-ARS, Gainesville, Florida, USA.

## Supplementary Data

79 Supplementary File 1

## References

[1] Le Gall O, Christian P, Fauquet CM, King AM, Knowles NJ (2008). *Picornavirales*, a proposed order of positive-sense single-stranded RNA viruses with a pseudo-T=3 virion architecture. Arch Virol.

[2] Valles SM, Strong CA, Hashimoto Y (2007). A new positive-strand RNA virus with unique genome characteristics from the red imported fire ant, Solenopsis invicta. Virology.

[3] Hashimoto Y, Valles SM (2008). Infection characteristics of Solenopsis invicta virus 2 in the red imported fire ant, Solenopsis invicta. J Invertebr Pathol.

[4] Valles SM, Varone L, Ramírez L, Briano J (2009). Multiplex detection of Solenopsis invicta viruses -1, -2, and -3. J Virol Methods.

[5] Valles SM, Oi DH, Porter SD (2010). Seasonal variation and the co-occurrence of four pathogens and a group of parasites among monogyne and polygyne fire ant colonies. Biological Control.

[6] Manfredini F, Shoemaker D, Grozinger CM (2016). Dynamic changes in host-virus interactions associated with colony founding and social environment in fire ant queens (Solenopsis invicta). Ecol Evol.

[7] Koonin EV, Wolf YI, Nagasaki K, Dolja VV (2008). The Big Bang of picorna-like virus evolution antedates the radiation of eukaryotic supergroups. Nat Rev Microbiol.

[8] Shi M, Lin XD, Tian JH, Chen LJ, Chen X (2016). Redefining the invertebrate RNA virosphere. Nature.

[9] Grabherr MG, Haas BJ, Yassour M, Levin JZ, Thompson DA (2011). Full-length transcriptome assembly from RNA-Seq data without a reference genome. Nat Biotechnol.

[10] Haas BJ, Papanicolaou A, Yassour M, Grabherr M, Blood PD (2013). De novo transcript sequence reconstruction from RNA-seq using the Trinity platform for reference generation and analysis. Nat Protoc.

[11] Zerbino DR, Birney E (2008). Velvet: algorithms for de novo short read assembly using de Bruijn graphs. Genome Res.

[12] Langmead B, Salzberg SL (2012). Fast gapped-read alignment with bowtie 2. Nat Methods.

[13] Söding J, Biegert A, Lupas AN (2005). The HHpred interactive server for protein homology detection and structure prediction. Nucleic Acids Res.

[14] Finn RD, Coggill P, Eberhardt RY, Eddy SR, Mistry J (2016). The Pfam protein families database: towards a more sustainable future. Nucleic Acids Res.

[15] Berman HM, Westbrook J, Feng Z, Gilliland G, Bhat TN (2000). The protein data bank. Nucleic Acids Res.

[16] Alva V, Nam SZ, Söding J, Lupas AN (2016). The MPI bioinformatics Toolkit as an integrative platform for advanced protein sequence and structure analysis. Nucleic Acids Res.

[17] Söding J (2005). Protein homology detection by HMM-HMM comparison. Bioinformatics.

[18] Krogh A, Larsson B, Von Heijne G, Sonnhammer EL (2001). Predicting transmembrane protein topology with a hidden Markov model: application to complete genomes. J Mol Biol.

[19] Koonin EV, Dolja VV (1993). Evolution and taxonomy of positive-strand RNA viruses: implications of comparative analysis of amino acid sequences. Crit Rev Biochem Mol Biol.

[20] Edgar RC (2004). MUSCLE: multiple sequence alignment with high accuracy and high throughput. Nucleic Acids Res.

[21] Ronquist F, Teslenko M, van der Mark P, Ayres DL, Darling A (2012). MrBayes 3.2: efficient bayesian phylogenetic inference and model choice across a large model space. Syst Biol.

[22] Isogai M, Tatuto N, Ujiie C, Watanabe M, Yoshikawa N (2012). Identification and characterization of blueberry latent spherical virus, a new member of subgroup C in the genus Nepovirus. Arch Virol.

[23] Gorbalenya AE, Koonin EV, Blinov VM, Donchenko AP (1988). Sobemovirus genome appears to encode a serine protease related to cysteine proteases of Picornaviruses. FEBS Lett.

[24] Gorbalenya AE, Donchenko AP, Blinov VM, Koonin EV (1989). Cysteine proteases of positive strand RNA viruses and chymotrypsin-like serine proteases. FEBS Lett.

[25] O'Reilly EK, Kao CC (1998). Analysis of RNA-dependent RNA polymerase structure and function as guided by known polymerase structures and computer predictions of secondary structure. Virology.

[26] Jablonski SA, Luo M, Morrow CD (1991). Enzymatic activity of poliovirus RNA polymerase mutants with single amino acid changes in the conserved YGDD amino acid motif. J Virol.

[27] Hong Y, Hunt AG (1996). RNA polymerase activity catalyzed by a potyvirus-encoded RNA-dependent RNA polymerase. Virology.

[28] Lohmann V, Körner F, Herian U, Bartenschlager R, Herian U, Ko F (1997). Biochemical properties of hepatitis C virus NS5B RNA-dependent RNA polymerase and identification of amino acid sequence motifs essential for enzymatic activity. J Virol.

[29] Sankar S, Porter AG (1992). Point mutations which drastically affect the polymerization activity of encephalomyocarditis virus RNA-dependent RNA polymerase correspond to the active site of Escherichia coli DNA polymerase I. J Biol Chem.

[30] Biswas SK, Nayak DP (1994). Mutational analysis of the conserved motifs of influenza A virus polymerase basic protein 1. J Virol.

[31] Sánchez AB, de La Torre JC, de TJC, Ab S (2005). Genetic and biochemical evidence for an oligomeric structure of the functional L polymerase of the prototypic Arenavirus lymphocytic choriomeningitis virus. J Virol.

[32] Zamoto-Niikura A, Terasaki K, Ikegami T, Peters CJ, Makino S (2009). Rift valley fever virus L protein forms a biologically active oligomer. J Virol.

[33] Te Velthuis AJ (2014). Common and unique features of viral RNA-dependent polymerases. Cell Mol Life Sci.

[34] Letzel T, Mundt E, Gorbalenya AE (2007). Evidence for functional significance of the permuted C motif in Co^2+^-stimulated RNA-dependent RNA polymerase of infectious bursal disease virus. J Gen Virol.

[35] Gorbalenya AE, Pringle FM, Zeddam JL, Luke BT, Cameron CE (2002). The palm subdomain-based active site is internally permuted in viral RNA-dependent RNA polymerases of an ancient lineage. J Mol Biol.

[36] Cook S, Chung BY, Bass D, Moureau G, Tang S (2013). Novel virus discovery and genome reconstruction from field RNA samples reveals highly divergent viruses in dipteran hosts. PLoS One.

[37] Guo H, Ingolia NT, Weissman JS, Bartel DP (2010). Mammalian microRNAs predominantly act to decrease target mRNA levels. Nature.

[38] Rice P, Longden I, Bleasby A (2000). EMBOSS: the European molecular biology open software suite. Trends Genet.

[39] Altschul SF, Gish W, Miller W, Myers EW, Lipman DJ (1990). Basic local alignment search tool. J Mol Biol.

[40] Kelley LA, Sternberg MJ (2009). Protein structure prediction on the web: a case study using the Phyre server. Nat Protoc.

