# Supplementary material for: Polycipiviridae: a proposed new family of polycistronic picorna-like RNA viruses
Source: J Gen Virol. 2017 Aug 31;98(9):2368–78. doi: 10.1099/jgv.0.000902 (PMC5656759; doi:10.1099/jgv.0.000902)
Supplement: Supplementary File 1 [file jgv-98-2368-s001.pdf]

(a)

```
                            ((((((((((   ))))))))))
MF041810  MsaV-1      AAUAAUUAUAUGGGGGCUACCCCCAUAUAUUUGAGUUCAUGCUACGGUGUGUUCCAAAC...
MF041809  LneV-1      ------------------ACCCCCAUAUUUUUGAGUUCAUGUUACGAUGUGUUCCAAAU...
                                        ********** ************ **** ***********
```

(b)

```
                                  (((((((((     )))))))))
MF041808  SINV-4      UUUAAAAAUAACCAUGUAGGGCCU-ACCCUACAUGUGCGUACAAAAGGGACGUAAUAUU...
KX883910  ShiV-8      -------------ACGUAGGGCCUACCCCUAUGUGUUGUUAU----------UAUUAUC...
LA866448  M.pha TSA   ---------------GUAGGGCCUUACCCUGCACGUUGUUGU----------UAUUAUU...
                                     *********  ****    **   *            ** ***
```

(c)

```
                      ((((((((      ))))))))
KX883910  ShiV-8      ACGUAGGGCCU-ACCCCUAUGUGUUGUUAUUAUUAUCUACGGAUUUUAAUACUUUA-UU...
LA866448  M.pha TSA   --GUAGGGCCUUA-CCCUGCACGUUGUUGUUAUUAUUUACGAAUUUUAACACUUUAAGU...
                        ********* * ****    ****** ******* **** ******* ******   *
```

(d)

```
                                      (((((((    )))))))
EF428566  SINV-2      UUUUAAAUAGAUCGCAUAUACAGACUCCUGUAUAGGGAGGGCAUCAUUGGUCGGCAUGG...

                                            (((((( ((((   ))))))))))
LI526777  L.neg TSA   UUUAUUUAACUUAAUAAUAGUUUCUGCUUGAUUGGCAAUCAGCAGAUUUAACAUCACUA...

                          ((((((((((            ))))))))))
KA182589  C.rip TSA   AAUAAAAGAACGCGUCCUCUUUCACGCGCGUUCUUUGAAUAGAAAUGGUAAUAUAGUAG...
```

Figure S1. Analysis of 5′ ends of polycipivirus sequences. (a) Alignment of 5′ ends of MF041810 (MsaV-1) and MF041809 (LneV-1). (b) Alignment of 5′ ends of MF041808 (SINV-4), KX883910 (Shuangao insect virus 8) and LA866448 (*Monomorium pharaonis* TSA). (c) Alignment of 5′ ends of KX883910 (Shuangao insect virus 8) and LA866448 (*Monomorium pharaonis* TSA). (d) Other polycipivirus sequences with possibly near-complete 5′ UTRs. We hypothesis that MF041808, EF428566 and LI526777 are within a few nucleotides of being 5′-complete, while MF041810, MF041809, KX883910 and LA866448 are missing just 5–30 nt of 5′ sequence. Predicted 5′-proximal stem-loops are highlighted in yellow with potential base-pairings indicated with parentheses. Substitutions from G:C or A:U, to G:U pairings are indicated in cyan. 5′-terminal A/U tracts are indicated in red.

```
                                    A                        B                      C
                      _________________     ____________        _________________
Hubei-82              MHIQFYSEPGAGKTDLS 47  YYGQ-KYMIIDE 32  DKGMLFNLNMVISNTNNPF
Ch. riparius TSA      FHVQFTSGPGYGKTDLS 45  YFAQ-NFGYLDE 32  DKGRMFQIRVLVSNTNNPY
Hubei-81C             FHVQLAGAPGIGKSSVL 31  FSSDVEKMIYDD 30  EKGTPFRVRVVGSATNVPY
Hubei-81I             FHVQLAGAPGIGKSSVL 31  FSSDVEKMIYDD 30  EKGTPFRVRVVGSATNVPY
SINV-4                FHIQFVGDAGVGKSKLT 32  YQQQ-KFMIIDD 30  EKGIQLNSDILVSSTNVPY
Shuangao-8            FHVQFVGKAGVGKSTMT 32  YQQQ-KFMIIDD 30  EKGIQLQSDILISSTNTPY
Mo. pharaonis TSA1    FHIQFVGAAGVGKSKLT 32  YQQQ-KFMIIDD 30  EKGIQLNSDILISSTNTPY
Fo. exsecta TSA       FHVQFVGEPGIGKSTLT 32  YSGD-KVMIIDD 30  DKGQTLDSDILISSTNVPY
LneV-1                FHVQFVGEPGIGKSTIT 32  YNGQ-TFMIIDD 30  DKGVHLDSDILISSTNIPY
MsaV-1                FHVQFVGEPGIGKSTIT 32  YNGQ-TYMIIDD 30  DKGVHLESDILISSTNMPY
Li. humile TSA        FHVQLCGAAGIGKSTLT 32  YAGQ-KIMIVDD 30  DKGCQLSSEIMISSTNTPY
La. neglectus TSA     FHIQFVGRPGIGKSTIT 32  YAGQ-RIMVVDD 30  DKGVQLTSEVLLSSTNTAY
LniV-1                FHVQFVGRPGIGKSTIT 32  YAGQ-KIMVVDD 30  DKGVQLTSEVLLSSTNTAY
Mo. pharaonis TSA2    FHIQLVGKPGIGKSTLT 32  YAGQ-KIMIADD 30  DKGVQLTSEIFLSSTNTAY
SINV-2                FHVQLVGRPGIGKSTLI 32  YAGQ-RIMIADD 30  DKGVQLTSEVFLSTTNTAY
                      :*:*: . .* **: :      :  :      *:      :**  :    :. * ** .:
```

Figure S2. Conserved helicase motifs in polycipivirus sequences. Polycipivirus helicases have all three conserved sequence motifs of picorna-like superfamily III helicases (as per Koonin and Dolja, 1993). Numbers indicate the lengths of intervening amino acids sequences not shown in the figure. Abbreviations: Hubei-82 – Hubei picorna-like virus 82; Hubei-81C/81I – Hubei picorna-like virus 81 from crustacea (C) or insects (I). Shuangao-8 – Shuangao picorna-like virus 8. *Mo. pharaonis* TSAs sequences are denoted as TSA1 for LA866448 and TSA2 for LA858223.

```
                           1              2                         3
Hubei-81C             QLFVLNQHSLR 36 PGKDLVFINCRDM 72 GGVPGGTSGSPCIGVGGTQG-RDLMGI--
Hubei-81I             QLFVLNQHSLR 36 PGKDLVFINCRDM 72 GGVPGGTSGSPCIGVGGTQG-RDLMGI--
SINV-4                QFLLVNRHMVE 33 PRSDAAIIFCRRF 69 GQTITGKSGAMLIVPNKASGHRNILGIQA
Shuangao-8            QFLILNRHMVE 32 PKSDVAIVFCRQL 69 GETVCGKSGSMLIVPNKASGNKNILGIQA
Mo. pharaonis TSA1    QFLILNRHMVE 32 PKSDVAIVFCRQL 69 GETVCGKSGSMLVVPNKISGHKNILGIQA
Fo. exsecta TSA       QFLIINAHTAD 32 PGNDLAIIFSRHL 68 GTTVAGKSGSMLVSPNKKPGHRNIVGIQA
LneV-1                QFLIINAHTAD 32 PNNDLAIIFSRHL 68 GATIAGKSGSMLMIPSRKPGHRSIVGIQA
MsaV-1                QFLIINSHTAD 32 PGNDLAIIFSRHL 68 GATVAGKSGSMLMIPSRKPGHRSIVGIQA
Li. humile TSA        QYLYVNKHVFL 33 DAGDLACIYSKSI 70 GTTVVGKSGSPVVYTRQISGSFAILGIQA
La. neglectus TSA     QYIILNKHVLK 33 PTGDLAIIYCRDL 67 GHTLGGRSGSTVLT--SLQSKPRIIGIQA
LniV-1                QYIILNKHVLK 33 PNGDLAIIYCRDL 67 GHTIGGRSGSTVLT--AIQAKPRIIGIQA
Mo. pharaonis TSA2    QYIILNKHVTK 30 PDGDLAIIWSRYV 67 GNTVLGNSGSTVVV--HHGSLTSILGIQA
SINV-2                QFVLCNSHIFD 32 KDRDLAIIFSRLF 67 GSTVSGRSGSPVIA--QVNGLARLIGIQS
Hubei-82              VFMVPN-HFLK 37 RGKDAALVALPKI 81 GDVRFGDSGSLVVHTNTKMQSHFLVGHMI
Ch. riparius TSA      IFMSCA-HTFA 36 DNKDIVLLYIEGF 66 VEVKPGDSGSLVMHDNPKIQNK-FIGL-I
                        .    *          * . :    .      .  * **:  :          ::*
```

Figure S3. Conserved protease motifs in polycipivirus sequences. Polycipivirus proteases contain sequence motifs of picorna-like proteases (as per Koonin and Dolja, 1993) including the catalytic triad of H, D and C/S. Unusually, polycipivirus sequences have S at the latter position whereas most members of the order *Picornavirales* have C. Virus name abbreviations are explained in the Figure S2 caption.

```
                         I                      II                       III              IV
Hubei-81C             FHAKDELR  9 KTRAIVCVNMAYNMCLRKYFGPFFSVMHK  8 MVGINPESITSHIM 11 ALDLSKFDSHVT
Hubei-81I             FHAKDELR  9 KTRAIVCVNMAYNMCLRKYFGPFFSVMHK  8 MVGINPESITSHIM 11 ALDLSKFDSHVT
SINV-4                DFPKDELR 16 KTRSVTCMSLDIILAWRRVTCDLFASLHR  8 APGMNPEGPDWGRL 11 DFDVSNWDGHMT
Shuangao-8            DFPKDELR 16 KTRSVTCMSLDIILAWRRVTCDLIASLHR  8 APGMNPEGPDWGRL 11 DFDVSNWDGHMT
Mo. pharaonis TSA1    DFPKDELR 16 KTRSVTCMSLDVILSWRRVTNDLFASLHR  8 APGMNPEGPDWGRL 11 DFDVSNWDGHMP
Fo. exsecta TSA       DFPKDELR 16 KTRSVTCMNMEFIFAWRRVTLDLFASLHR  8 GPGINPEGPDWTRL 11 DFDVSNWDGHMP
LneV-1                DFPKDELR 16 KTRSVTCMNMEFIFSWRRVTLDLFASLHR  8 GPGINPEGPDWTRL 11 DFDVSNWDGHMP
MsaV-1                DFPKDELR 16 KTRSVTCMNMEFIFAWRRVTLDLFASLHR  8 GPGINPEGPDWTRL 11 DFDVSNWDGHMP
Li. humile TSA        DFPKDELR 16 KTRSVTCMNMMYVLLWRELTLDMWAAFHR  8 CPGINPEGPEWSSA 11 DFDVSNWDGYFP
La. neglectus TSA     DFPKDELR 14 KTRSVTCMNVFYILAWRQLTLDFWASMHR  8 CPGINPEGPDWNNL 11 DFDVSNWDGFLR
LniV-1                DFPKDELR 14 KTRSVTCMNVFYILAWRQLTLDFWASMHR  8 CPGINPEGPDWNNL 11 DFDVSNWDGFLR
Mo. pharaonis TSA2    DFPKDELR 14 KTRSVTCMNVYYILAWRRYTLKFWSAMHR  8 CPGINPEGPEWNNL 11 DFDVSNWDGFLF
SINV-2                DFPKDELR 14 RTRSVTCMNVYYILAWRRYTMRFWSAMHR  8 GPGINPEGPEWSAL 11 DFDVSNWDGFLF
Hubei-82              EFIKKELV  8 KTRTVGTGNIIHQIVYNKCNKALQLLVKN 10 AMGLDLE-IHGNQI 11 DFDVKAWEASIN
Ch. riparius TSA      EFTKKELV  8 KTRTVATGNMIHQIVYNKLFKHLYILFKN 10 ALGVDPV-RHWDQI 11 DFDVKAWEEKVN
                       . *.**     .**::   .:   :  .     :   .:.      *::              :*:. ::  .

                                      V                      VI             VII         VIII
Hubei-81C             46 GMKSGAAIVAEINTLVHWIVFLAMYY 21 TLFLYGDDMICSI 45 EYQTFLKR 15 IDKSVINDL
Hubei-81I             46 GMKSGAAIVAEINTLVHWIVFLAMYY 21 TLFLYGDDMICSI 45 DYQTFLKR 15 IDKTVINDL
SINV-4                50 GLISGFPGTAETNTLAHILLFYYFYL 21 HAIFYGDDVQASI 41 DSQ-FLKS 13 LDINVVYDM
Shuangao-8            50 GLISGFPGTAEVNTLAHLILFYYYYL 21 HSIFYGDDVQASI 41 DSQ-FLKS 13 LDISVVYDM
Mo. pharaonis TSA1    50 GLISGFPGTAETNTLAHLILFYYYYL 21 HSIFYGDDVQASI 41 ESQ-FLKS 13 LDISVIYDM
Fo. exsecta TSA       50 GLVSGFPGTAEINTLVHLILMYYFYL 21 SPIFYGDDVLISI 41 DCQ-FLKS 13 MDVSVCYDL
LneV-1                50 GLISGFPGTAEVNTLVHLILMYYFYL 21 SPVFYGDDVIMSV 41 DCQ-FLKS 13 MDISVVYDL
MsaV-1                50 GLISGFPGTAEVNTLVHLILMYYFYL 21 SPMFYGDDVIISI 41 DCQ-FLKS 13 MDISVVYDL
Li. humile TSA        50 GMISGFPGTAELNSMAHLILIIYIYL 19 RCLIYGDDIILSF 41 QCQ-FLKS 13 MELSVAYDL
La. neglectus TSA     48 GLVSGFPGTAEINTLSHWLLILYIYL 18 SVAIYGDDIILTF 41 KCT-FLKS 13 MDEEVLNNL
LniV-1                48 GLVSGFPGTAEINTLSHWLLILYIYL 18 SVAIYGDDIILTF 41 KCT-FLKS 13 MDEEVLNNL
Mo. pharaonis TSA2    50 GLVSGFPGTAEVNTLVHWLLYICIYL 18 SVAIYGDDIIITF 41 DCK-FLKS 13 MDMDVAHDL
SINV-2                50 GIISGFPGTAEVNTLAHILLIVCIYL 18 SAILYGDDILLTI 41 QCQ-FLKS 13 LDLEVAYDL
Hubei-82              56 GLLSGHPGTFIENTDVHIMLVYLIAR 21 KVVVAADDIVMAI 39 EIQ-FLKH 13 PLTSIIYQL
Ch. riparius TSA      56 GLLSGHPGTLMENSEIHTMIMYLICF 20 RFILAADDIVIAI 39 EIQ-FLKQ 12 PNWDIIYQL
                         *: ** . .   *:  * ::             . .**:  :.    .   ***         :  ::
```

Figure S4. Conserved RdRp motifs in polycipivirus sequences. Polycipivirus RdRps have all eight picorna-like superfamily I RdRp sequence motifs (as per Koonin and Dolja, 1993). Unusually, two sequences have a G to A substitution at the normally highly conserved GDD sequence in motif VI. Virus name abbreviations are explained in the Figure S2 caption.
